# Southern Dental Association

**Published:** 1869-09

**Authors:** 


### SOUTHERN DENTAL ASSOCIATION.

In obedience to the call issued sometime previous, a very considerable number of the profession of the South met at Atlanta, Ga., July 28th, for the purpose of organizing a Southern Dental Association. We are pleased to learn, that the object aimed at was readily and harmoniously accomplished—that an Association was organized upon a good basis, and in such a manner as to promise much for the profession in that part of the country. Though this work has been delayed somewhat, we doubt not, indeed we know from the ability of many engaged in it, that it will not be behind many of the older societies in efficient work.

The next meeting will be held in New Orleans, in April next, when we hope there will be a very large attendance of earnest workers.

The officers are Dr. W. T. Arrington, Memphis, Tenn., President. Dr.———, New Orleans, Rec. Secretary.

